# Supplementary material for: The Blind Watchmaker Network: Scale-Freeness and Evolution
Source: PLoS One. 2008 Feb 27;3(2):e1690. doi: 10.1371/journal.pone.0001690 (PMC2246025; doi:10.1371/journal.pone.0001690)
Supplement: Text S1 — (0.05 MB PDF) [file pone.0001690.s001.pdf]

1 Supporting Information

The combinatorics for the CBB-model goes as follows: You first fill all the N boxes with one ball and then you fill in the remaining M-N balls without any further restriction. The total number of different ways then becomes

$$\Omega = \frac{M!}{\Pi_{k=1} N(k)! \Pi_{k=1} k^{N(k)}} \tag{1}$$

Using the Stirling approximation the entropy $S = \ln \Omega$ takes the form

$$S = M \ln M - N \ln N - N \sum_{k=1} n(k) \ln kn(k) \tag{2}$$

where *n(k)=N(k)/N*. Variational calculus provides a method for finding the distribution *n(k)* which maximizes S (and hence also Ω). Since N and M are constant, we want to the maximize $s[n(k)] = -\sum_{k=1} n(k) \ln kn(k)$ with respect to all possible distributions *n(k)* subject to the two constraints $\sum_{k=1} n(k) = 1$ and $\sum_{k=1} kn(k) = \langle k \rangle = M/N$. The constraints are implemented by two Lagrangian multipliers a and b which means that the functional to be maximized is given by

$$g[n(k)] = -\sum_{k=1} n(k) \ln kn(k) - a \sum_{k=1} n(k) - b \sum_{k=1} kn(k) \tag{3}$$

The condition for the maximum is $\frac{\delta g[n(k)]}{\delta n(k)} = 0$ and leads to the equation $\ln kn(k) + 1 + a + bk = 0$ which has the solution $n(k) = A \exp(-bk)/k$. The value of the constants A and b follows from the two constraints $\sum_{k=1} n(k) = 1$ and $\sum_{k=1} kn(k) = \langle k \rangle$. In Fig. 2(a) of the main text, this solution is shown to be identical to the corresponding algorithm solution.

Random with respect to the relevant states instead corresponds to the maximum of $\widetilde{S} = \ln \widetilde{p}\Omega = S - S_a = S - \sum_{k=1} N(k) \ln k$. The functional to maximizes is

$$\widetilde{g}[n(k)] = g[n(k)] - \sum_{k=1} n(k) \ln k \tag{4}$$

and has the solution $n(k) = A \exp(-bk)/k^2$. In Fig. 2(b) of the main text, this variational solution is shown to be identical to the corresponding algorithm solution.

The algorithm which also includes the network constraints goes as follows:

1) pick two boxes (nodes) A and B randomly with probability $p \sim k^2$.

2) pick a random ball in A and move to B.

3) If the attempted move is forbidden by a constraint choose another ball in A. Repeat until one ball is moved. Then choose two new boxes (nodes).

4) If no ball can be moved from A, choose two new boxes (nodes).

The network constraints are introduced through step 3 in the algorithm in such away as to ensure the least possible constraining effect.

A notable difference between the real metabolic networks and the corresponding blind watchmaker network is the number of nodes with just a single

link: the number of single link nodes for the metabolic networks is only about 20% of the number for the corresponding blind watchmaker network. In order to investigate how important the number of single-link nodes are for the global statistical properties of the network structure, we introduce an additional constraint into the blind watchmaker network: the average number of single-link nodes are constrained to be the same as for the metabolic networks. Again we choose a constraint which achieves this in an unbiased way. The constraint is again introduced into step 3 of the above algorithm and takes the form of an upper limit: The number of single-link nodes can never exceed a maximum number $N_{\max}(k = 1)$. Any move which violates this condition is forbidden. $N_{\max}(k = 1)$ is adjusted so as to give the same average $\langle n(k = 1)\rangle$ as the metabolic networks. This additional constraint also increases the power-law exponent slightly, from $\gamma \approx 2.1$ to $\gamma \approx 2.2$. The result is presented in Figs 3e and f in the main text. As seen the agreement between the blind watchmaker network and the metabolic networks is now extraordinary. This shows that the number of single-link nodes is not an insignificant detail but a decisive factor reflected in the global statistical properties of the network.
